# Impact of interval after fresh cycle cancellation on first frozen embryo transfer outcomes

**DOI:** 10.3389/fendo.2026.1840194

**Published:** 2026-05-25

**Authors:** Xiaoyue Peng, Hailong Li, Xinna Huang, Ying Zhang, Zhifeng Sun

**Affiliations:** 1 Reproductive Medicine Center, Renmin Hospital, Hubei University of Medicine, Shiyan, Hubei Province, China; 2 Hubei clinical research center for reproductive medicine, Shiyan, Hubei Province, China; 3 Biomedical Engineering College, Hubei University of Medicine, Shiyan, China; 4 Healthcare Big Data Center, School of Public Health, Hubei University of Medicine, Shiyan, China; 5 Shiyan Key Laboratory of Reproduction and Genetics, Renmin Hospital, Hubei University of Medicine, Shiyan, Hubei Province, China

**Keywords:** fresh cycle cancellation, frozen embryo transfer, IVF, pregnancy outcomes, time interval

## Abstract

**Background:**

Frozen embryo transfer (FET) has become an integral component of assisted reproductive technology (ART), particularly after cancellation of fresh embryo transfer cycles. However, the optimal timing of FET after fresh cycle cancellation remains unclear, with conflicting evidence as to whether shorter or longer intervals are associated with better pregnancy outcomes. Previous studies have largely overlooked age-specific effects, despite established differences in endometrial receptivity and ovarian reserve between younger and older women. This study aimed to evaluate the association between the interval from oocyte retrieval to FET and reproductive outcomes after fresh cycle cancellation, stratified by maternal age, to provide evidence-based guidance for clinical decision-making.

**Methods:**

This retrospective cohort study included women undergoing their first FET after cancellation of fresh embryo transfer following *in vitro* fertilization/intracytoplasmic sperm injection at a single center between January 2018 and December 2023. Participants were categorized according to the interval from oocyte retrieval to FET: ≤3 months (n = 1,011), 3–6 months (n = 529), and ≥6 months (n = 297). Subgroup analyses were performed by maternal age. The primary outcomes were live birth rate (LBR), clinical pregnancy rate (CPR), and clinical pregnancy loss. Multivariable logistic regression was used to adjust for potential confounders.

**Results:**

A total of 1,837 women were included. Among women aged <35 years, longer intervals to FET (3–6 months and ≥6 months) were associated with lower odds of clinical pregnancy (adjusted odds ratio [AOR], 0.77; 95% confidence interval [CI], 0.57–1.05 and 0.69; 95% CI, 0.47–1.00, respectively) and live birth (AOR, 0.72; 95% CI, 0.54–0.95 and 0.71; 95% CI, 0.50–1.02, respectively) compared with an interval of ≤3 months. Among women aged 35–40 years, an interval of ≥6 months was associated with a significantly increased risk of clinical pregnancy loss (AOR, 2.11; 95% CI, 1.10–3.96).

**Conclusions:**

In women younger than 35 years, undergoing FET within 3 months after fresh cycle cancellation may be associated with more favorable reproductive outcomes. In women aged 35–40 years, delaying FET for ≥6 months may be associated with an increased risk of clinical pregnancy loss. These findings support an age-stratified approach to FET timing after fresh cycle cancellation.

## Introduction

Since the introduction of *in vitro* fertilization (IVF), frozen embryo transfer (FET) has become an integral component of assisted reproductive technology (ART) (1). Although previous studies have extensively compared FET with fresh embryo transfer, less attention has been paid to the optimal timing of FET within a freeze-all approach after cancellation of fresh embryo transfer. Determining the appropriate interval between fresh cycle cancellation and subsequent FET remains a clinically relevant yet insufficiently investigated question, particularly in the context of age-stratified outcomes.

Existing studies have largely focused either on comparisons between FET and fresh embryo transfer or on freeze-all strategies without specifically addressing the timing of FET after fresh cycle cancellation (2, 3). Some retrospective studies have suggested that immediate FET, typically performed within the first menstrual cycle, results in live birth rates comparable to those of delayed transfer (4), whereas others have proposed that a longer interval may be beneficial by allowing adequate endometrial recovery (5). In addition, multicenter randomized controlled trials have reported improved ongoing pregnancy or live birth outcomes with immediate FET (6, 7), while a large retrospective study found no significant differences in live birth rates across different transfer intervals (8, 9). However, these studies frequently involved heterogeneous populations and often did not specifically evaluate women whose fresh embryo transfer was canceled because of clinical considerations such as ovarian hyperstimulation syndrome (OHSS) risk or suboptimal endometrial conditions.

Importantly, the effect of the interval to FET may differ according to maternal age, which is closely associated with endometrial receptivity, embryo competence, and the risk of pregnancy loss (10). Women of advanced reproductive age (≥35 years) face distinct reproductive challenges, including diminished ovarian reserve and an increased likelihood of embryo aneuploidy, which may make them more vulnerable to the adverse effects of delayed transfer (11, 12). In contrast, younger women may derive greater benefit from shorter intervals because of more rapid physiological recovery after ovarian stimulation. Despite these biologically plausible differences, age-stratified evidence in this specific clinical setting remains limited.

Therefore, this study aimed to investigate the association between the interval from oocyte retrieval to the first FET and pregnancy outcomes in women undergoing their first FET after fresh cycle cancellation, with stratified analyses according to maternal age (<35 vs. 35–40 years). Using data from 1,837 women, we examined whether shorter (≤3 months), intermediate (3–6 months), and prolonged (≥6 months) intervals were associated with differences in live birth rate (LBR), clinical pregnancy rate (CPR), and clinical pregnancy loss. We anticipated that these findings would provide clinically relevant evidence to support individualized decision-making regarding the timing of FET after fresh cycle cancellation.

## Materials and methods

### Study population and design

**Figure 1:**
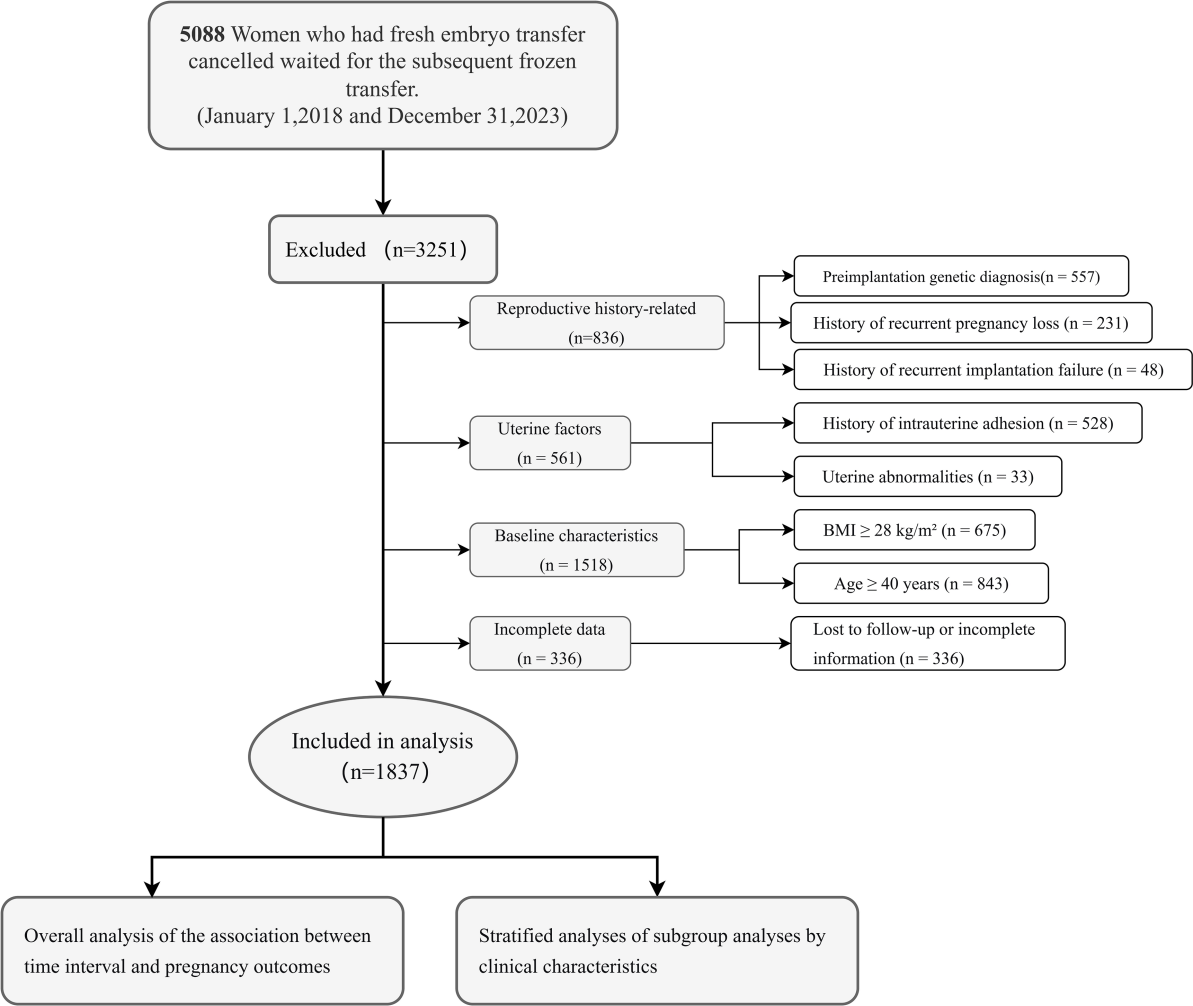
Flow diagram for the study population.

This retrospective study included women who underwent assisted reproductive technology (ART) treatment at the Reproductive Medicine Center of Shiyan Renmin Hospital between January 2018 and December 2023. Clinical data were collected with written informed consent from all women and approval from the hospital ethics committee (SYSRMYY-2025-025). A flow diagram of the study population is presented in **Figure 1**. The study included 1,837 women who underwent FET after cancellation of fresh embryo transfer during their first stimulated ART cycle. The exclusion criteria were as follows (1): recurrent pregnancy loss, defined as two or more consecutive pregnancy losses before 28 weeks of gestation with the same partner (13) (2); recurrent implantation failure, defined as failure to achieve a clinical pregnancy after transfer of at least four good-quality embryos over a minimum of three fresh or frozen embryo transfer cycles in women aged <40 years (14) (3); uterine abnormalities, including congenital uterine anomalies (e.g., bicornuate uterus or unicornuate uterus) or a history of intrauterine adhesions (4); transfer of embryos that had undergone preimplantation genetic testing (5); transfer of embryos derived from oocyte donation or cryopreserved oocytes (6); body mass index (BMI) ≥28 kg/m² (7); age ≥40 years; and (8) loss to follow-up.

### Definition of study group

The interval was defined as the time from oocyte retrieval to the first subsequent frozen embryo transfer (FET). Participants were categorized into three groups according to this interval: ≤3 months (n = 1,011), 3–6 months (n = 529), and ≥6 months (n = 297). Participants were further stratified by maternal age (<35 years and 35–40 years), and the association between the interval to FET and pregnancy outcomes was evaluated within each age subgroup.

The interval categories were primarily based on standard clinical practice in FET cycles. After oocyte retrieval, the first menstrual cycle typically occurs within 7–10 days, followed by a second natural cycle of approximately 30 days. For women requiring down-regulation, an additional 35 days is generally required, followed by approximately 21 days for endometrial preparation. Collectively, these treatment timelines correspond to an interval of approximately 90 days, which served as the basis for the ≤3-month group. The 3–6-month and ≥6-month groups were subsequently defined according to clinically relevant timeframes to facilitate comparison across cohorts.

### Outcome definitions

The primary outcome was live birth, defined as delivery of at least one live infant after 28 weeks of gestation. Secondary outcomes included conception, clinical pregnancy, clinical pregnancy loss, singleton live birth, and twin live birth. Conception was defined as a positive pregnancy test with a serum human chorionic gonadotropin level of ≥50 IU/L at 14 days after FET. Clinical pregnancy was defined as the presence of one or more intrauterine and/or extrauterine gestational sacs detected by ultrasonography. Clinical pregnancy loss was defined as pregnancy loss, including early and late miscarriage, occurring before 28 weeks of gestation.

### Statistical analysis

Continuous variables are presented as mean ± standard deviation (SD) for normally distributed data and median (interquartile range [IQR]) for non-normally distributed data. Categorical variables are presented as number (percentage). Normality was assessed using the Shapiro-Wilk test. Comparisons among the three interval groups were performed using analysis of variance (ANOVA) or the Kruskal-Wallis test for continuous variables, and the chi-square test or Fisher’s exact test for categorical variables. In stratified analyses, categorical variables were compared using the Cochran-Mantel-Haenszel test.

To account for potential confounding, multivariable logistic regression models were constructed. Variables showing significant between-group differences in baseline characteristics, together with clinically relevant covariates, were included in the adjusted models. The following variables were included: female age; body mass index (BMI); type of infertility (primary vs. secondary); duration of infertility; gravidity (0, 1, or ≥2); previous abortion (0, 1, or ≥2); total antral follicle count (AFC); anti-Müllerian hormone (AMH) level; number of retrieved oocytes; fertilization method (IVF vs. ICSI); endometrial preparation method for FET (down-regulation endometrial preparation vs. HRT); stage of transferred embryos (D3 vs. D5); embryo score category (AA/AB/BA vs. BB vs. other) (15); number of viable embryos; number of high-quality embryos; and number of transferred embryos (1 vs. 2). Adjusted odds ratios (AORs) and 95% confidence intervals (CIs) were calculated to evaluate the association between the interval to FET and pregnancy outcomes.

All statistical analyses were performed using R software, version 4.4.2 (R Foundation for Statistical Computing, Vienna, Austria). All tests were two-sided, and P < 0.05 was considered statistically significant.

## Results

Between January 2018 and December 2023, a total of 1,837 women who met the inclusion criteria were included in this study. Among them, 1,011 (55.0%), 529 (28.8%), and 297 (16.2%) women were assigned to the ≤3-month, 3–6-month, and ≥6-month groups, respectively. Participants were further stratified by maternal age. The <35-year subgroup included 1,235 women, of whom 667 (54.0%), 376 (30.4%), and 192 (15.5%) were classified into the ≤3-month, 3–6-month, and ≥6-month groups, respectively. The 35–40-year subgroup included 602 women, with 344 (57.1%), 153 (25.4%), and 105 (17.4%) women in the corresponding interval groups.

Baseline characteristics of the study population are presented in **Table 1**. Among the three interval groups, no significant differences were observed in female age, male age, duration of infertility, previous abortion, fertilization method, or number of transferred embryos. In contrast, body mass index (BMI), type of infertility, gravidity, total antral follicle count (AFC), anti-Müllerian hormone (AMH) level, number of retrieved oocytes, endometrial preparation method, stage of transferred embryos, number of viable embryos, number of high-quality embryos, and embryo score category differed significantly among the groups. These variables were subsequently accounted for in the multivariable regression analyses.

**Table 1:** Baseline characteristics of the overall data.

Characteristic	≤3 months	3–6 months	≥6 months	P value
	(n=1011)	(n=529)	(n=297)	
**Female age (IQR)**	32(29-36)	32(29-35)	33(29-36)	0.25
**Male age (IQR)**	33(30-37)	33(30-37)	33(30-37)	0.81
**BMI(IQR)**	22.39(20.46-24.44)	22.37(20.45-24.34)	22.77(21.03-25.10)	0.024
Types of infertility				
Primary infertility	451(44.6%)	277(52.4%)	146(49.2%)	0.013
Secondary infertility	560(55.4%)	252(47.6%)	151(50.8%)	
Duration of infertility (IQR)	3(1.5-5)	3(1.5-5)	3(2.0-6)	0.15
Gravidity				
0	439(43.4%)	268(50.7%)	139(46.8%)	0.012
1	241(23.8%)	114(21.6%)	83(27.9%)	
≥2	331(32.7%)	147(27.8%)	75(25.3%)	
Previous abortion				
0	649(64.2%)	359(67.9%)	193(65.0%)	0.57
1	252(24.9%)	112(21.2%)	73(24.6%)	
≥2	110(10.9%)	58(11.0%)	31(10.4%)	
Total AFC (IQR)	7(6-10)	8(6-11)	6(5-10)	<0.001
AMH (IQR)	2.1(1.42-3.31)	2.28(1.51-3.83)	1.85(1.15-3.08)	<0.001
No. of retrieved oocytes (IQR)	6(5-9)	7(5-9)	5(4-8)	<0.001
Fertilization method				
IVF	843(83.4%)	436(82.4%)	251(84.5%)	0.73
ICSI	168(16.6%)	93(17.6%)	46(15.5%)	
Endometrial preparation method				
De-HRT	952(94.2%)	500(94.5%)	267(89.9%)	0.018
HRT	59(5.8%)	29(5.5%)	30(10.1%)	
The stage of transferred embryos				
D3	106(10.5%)	65(12.3%)	58(19.5%)	<0.001
D5	905(89.5%)	464(87.7%)	239(80.5%)	
**No. of viable embryos**	3(2-4)	3(2-4)	2(1-4)	<0.001
**No. of high-quality embryos (IQR)**	2(1-3)	2(1-3)	1(0-3)	<0.001
No. of transferred embryos				
1	400(39.6%)	187(35.3%)	116(39.1%)	0.29
2	611(60.4%)	342(64.7%)	181(60.9%)	
Scores of transferred embryos				
AA, AB, BA	441(43.6%)	248(46.9%)	113(38%)	<0.001
BB	439(34.4%)	201(38.0%)	118(39.7%)	
other	131(13.0%)	80(15.1%)	66(22.2%)	

Bold values indicate statistically significant differences (P < 0.05).

### Overall outcomes

**Table 2** presents the association between interval categories and pregnancy outcomes. The observed difference in twin live birth rates (P = 0.04) may be attributable to variation in the number of embryos transferred across groups. After adjustment for this potential confounder, no statistically significant association was observed between interval category and reproductive outcomes.

**Table 2:** Association between interval categories and overall pregnancy outcomes.

Outcome	≤3 months	3–6 months	≥6 months	P value
	(n=1011)	(n=529)	(n=297)	
Live Birth	582(57.6%)	286(54.1%)	149(50.2%)	0.06
Singleton	412(40.8%)	202(38.2%)	117(39.4%)	0.61
Twin	170(16.8%)	84(15.9%)	32(10.8%)	0.04
Conception	744(73.6%)	380(71.8%)	203(68.4%)	0.2
Clinical Pregnancy	683(67.6%)	345(65.2%)	184(62%)	0.18
Clinical Pregnancy Loss	94(13.8%)	52(15.1%)	32(17.4%)	0.75

### Age-stratified outcomes

Pregnancy outcomes stratified by maternal age are presented in **Table 3**. Among women aged <35 years, significant between-group differences were observed in live birth rate (P = 0.007) and clinical pregnancy rate (P = 0.008). In women aged 35–40 years, no significant between-group differences were observed in the unadjusted pregnancy outcomes. Multivariable logistic regression analyses are presented in **Tables 4**, **5**.

**Table 3:** Stratified analysis of the relationship between time intervals and pregnancy outcomes.

	<35 years				35–40 years			
	≤3 months	3–6 months	≥6 months	P value	≤3 months	3–6 months	≥6 months	P value
n	667	376	192		344	153	105	
Live Birth	428(64.2%)	218(58%)	105(54.7%)	0.007	154(44.8%)	68(44.4%)	44(41.9%)	0.64
Singleton	283(42.4%)	146(38.8%)	77(40.1%)	0.37	129(37.5%)	56(36.6%)	40(38.1%)	0.97
Twin	145(21.7%)	72(19.1%)	28(14.6%)	0.28	25(7.3%)	12(7.8%)	4(3.8%)	0.32
Conception	517(77.5%)	282(75%)	137(71.4%)	0.07	227(66%)	98(64.1%)	66(62.9%)	0.52
Clinical Pregnancy	482(72.3%)	257(68.4%)	120(62.5%)	0.008	201(58.4%)	88(57.5%)	64(61%)	0.73
Clinical Pregnancy Loss	53(11%)	33(12.8%)	13(10.8%)	0.79	41(20.4%)	19(21.6%)	19(29.7%)	0.14

**Table 4:** Stratified analyses of the association between categorical intervals and pregnancy outcomes.

(age<35 years)					
Age<35 years	≤3 months	3–6 months		≥6 months	
Outcome	OR (95% CI)	OR (95% CI)	P value	OR (95% CI)	P value
Live Birth					
Crude	1	0.77(0.59-1.01)	0.048	0.67(0.49-0.93)	0.017
Adjusted	1	0.72(0.54-0.95)	0.02	0.71(0.50-1.02)	0.063
Singleton					
Crude	1	0.86(0.66-1.11)	0.26	0.91(0.65-1.26)	0.57
Adjusted	1	0.81(0.57-1.13)	0.2	0.94(0.67-1.32)	0.72
Twin					
Crude	1	0.85(0.62-1.17)	0.32	0.61(0.39-0.94)	0.031
Adjusted	1	0.81(0.57-1.13)	0.22	0.66(0.40-1.06)	0.096
Conception					
Crude	1	0.87(0.65-1.17)	0.36	0.72(0.51-1.04)	0.079
Adjusted	1	0.81(0.59-1.12)	0.21	0.79(0.53-1.18)	0.24
Clinical Pregnancy					
Crude	1	0.83(0.63-1.09)	0.18	0.64(0.46-0.90)	0.0095
Adjusted	1	0.77(0.57-1.05)	0.094	0.69(0.47-1.00)	0.05
Clinical Pregnancy Loss					
Crude	1	1.11(0.7-1.74)	0.64	0.84(0.43-1.53)	0.59
Adjusted	1	1.10(0.69-1.75)	0.68	0.89(0.44-1.65)	0.71

**Table 5:** Stratified analyses of the association between categorical intervals and pregnancy outcomes.

(Age 35–40 years)					
Age ≥35 years	≤3 months	3–6 months		≥6 months	
Outcome	OR (95% CI)	OR (95% CI)	P value	OR (95% CI)	P value
Live Birth					
Crude	1	0.99(0.67-1.45)	0.95	0.89(0.57-1.38)	0.61
Adjusted	1	1.00(0.68-1.51)	0.96	1.06(0.66-1.70)	0.8
Singleton					
Crude	1	0.96(0.65-1.42)	0.85	1.03(0.65-1.60)	0.91
Adjusted	1	0.99(0.65-1.48)	0.94	1.17(0.72-1.89)	0.51
Twin					
Crude	1	1.09(0.51-2.18)	0.82	0.51(0.15-1.34)	0.22
Adjusted	1	0.93(0.3-2.08)	0.86	0.61(0.16-1.92)	0.44
Conception					
Crude	1	0.92(0.62-1.37)	0.68	0.87(0.56-1.38)	0.56
Adjusted	1	0.99(0.65-1.53)	0.98	1.13(0.69-1.86)	0.64
Clinical Pregnancy					
Crude	1	0.96(0.66-1.42)	0.85	1.11(0.71-1.74)	0.65
Adjusted	1	1.03(0.68-1.55)	0.89	1.46(0.90-2.39)	0.13
Clinical Pregnancy Loss					
Crude	1	1.05(0.58-1.85)	0.88	1.64(0.89-2.92)	0.11
Adjusted	1	1.15(0.62-2.06)	0.64	2.11(1.10-3.96)	0.021

Among women aged <35 years, compared with an interval of ≤3 months, longer intervals (3–6 months and ≥6 months) were associated with lower odds of clinical pregnancy (AOR, 0.77; 95% CI, 0.57–1.05 and AOR, 0.69; 95% CI, 0.47–1.00, respectively) and live birth (AOR, 0.72; 95% CI, 0.54–0.95 and AOR, 0.71; 95% CI, 0.50–1.02, respectively).

Among women aged 35–40 years, an interval of ≥6 months was associated with a higher risk of clinical pregnancy loss (AOR, 2.11; 95% CI, 1.10–3.96).

## Discussion

In this retrospective study, among women younger than 35 years, a longer interval between fresh embryo transfer cancellation and subsequent FET was associated with lower clinical pregnancy and live birth rates. Among women aged 35–40 years, prolonged delay before FET may be associated with an increased risk of clinical pregnancy loss.

Current evidence increasingly supports the clinical advantages of FET, including greater flexibility in transfer scheduling, avoidance of ovarian hyperstimulation syndrome (OHSS), and the opportunity to incorporate preimplantation genetic testing (PGT) when indicated (16). One plausible explanation is that the supraphysiological hormonal milieu associated with ovarian stimulation may impair endometrial receptivity, thereby reducing implantation potential in fresh transfer cycles (17, 18). Although some women prefer to delay FET to avoid potential complications or for personal reasons, prolonged waiting may adversely affect treatment continuity and psychological well-being. Previous studies have suggested that extended delays may increase anxiety and treatment discontinuation, which could indirectly influence reproductive outcomes (19, 20). Nevertheless, the optimal interval between oocyte retrieval and subsequent FET remains controversial. Several retrospective studies in elective freeze-all cycles have reported no significant association between transfer interval and reproductive outcomes (2, 3, 21), and a larger retrospective study with subgroup analyses reached a similar conclusion (8). In addition, a study conducted in natural-cycle FET reported that single blastocyst transfer performed in the first versus second menstrual cycle after a freeze-all strategy did not differ in pregnancy or perinatal outcomes (22). However, these studies differed in patient selection, cycle characteristics, and clinical protocols, which may partly explain the inconsistency across reports.

Notably, randomized controlled trials have provided evidence favoring earlier FET in certain settings. A multicenter randomized controlled trial reported higher ongoing pregnancy and live birth rates with immediate FET than with delayed FET in women with previous unsuccessful IVF-ET attempts (6). Another randomized controlled trial reached a similar conclusion, regardless of whether cleavage-stage or blastocyst-stage embryos were transferred (7). In addition, a retrospective study conducted under a non-selective freeze-all strategy found that FET during the first menstrual cycle was associated with a higher likelihood of live birth (4), which is broadly consistent with our findings in younger women. By contrast, the limited evidence supporting delayed FET is derived primarily from specific stimulation protocols, such as the long GnRH-agonist protocol, and was not fully adjusted for potential confounding factors (5).

In the present study, among women younger than 35 years, longer intervals to FET were associated with lower clinical pregnancy and live birth rates. One possible explanation is that, in younger women, endometrial recovery and restoration of reproductive endocrine balance after ovarian stimulation may occur relatively rapidly, such that unnecessary delay does not confer additional benefit and may instead reduce treatment efficiency (23). It is also possible that shorter waiting periods help reduce psychological burden and improve treatment adherence, although these factors were not directly assessed in the present study (19, 20). Further studies incorporating molecular markers of endometrial receptivity, ovarian hormonal dynamics, and embryo-endometrial interactions may help clarify the biological mechanisms underlying these interval-dependent associations (11, 24).

Among women aged 35–40 years, our findings suggest that delaying FET for ≥6 months may be associated with a higher risk of clinical pregnancy loss. Several mechanisms may underlie this observation. From an immunological perspective, older women exhibit distinct immune profiles, including higher proportions of effector memory CD4+ T cells, terminally differentiated CD4+ T cells, and mature natural killer (NK) cells than younger women (25). Dysregulation of CD4+ T-cell and NK-cell function has been implicated in the pathogenesis of pregnancy loss and may contribute to the increased vulnerability observed in older women (26). In addition, advanced maternal age is associated with a greater risk of chromosomal abnormalities, declining oocyte quality, and impaired endometrial receptivity, all of which may contribute to pregnancy loss (10, 11). Previous evidence has also shown age-related declines in clinical and ongoing pregnancy rates, further supporting the biological plausibility of our findings (27).

This study has several strengths. First, unlike previous studies that pooled women across age groups, we performed age-stratified analyses and found that the association between FET timing and reproductive outcomes differed by maternal age. Second, we focused specifically on women undergoing their first FET after fresh embryo transfer cancellation, a clinically distinct population that has often been underrepresented in broader freeze-all studies. Third, the relatively large sample size of 1,837 participants increased the statistical robustness of our analyses and improved the reliability of the age-specific findings.

Several limitations of this study should be acknowledged. First, the uneven distribution of participants across the longer interval groups may have reduced statistical power for detecting modest associations, particularly in subgroup analyses. Second, because of the retrospective study design, the findings remain susceptible to selection bias, information bias, and residual confounding. Although multivariable regression was used to adjust for measured confounders, the influence of unmeasured variables cannot be excluded. In addition, the specific reasons for delayed FET, including medical, logistical, and patient-driven factors, were not consistently recorded in the retrospective dataset; therefore, we were unable to distinguish between active and passive delay. Furthermore, some potentially relevant clinical factors, such as detailed endometriosis-related treatment history and prior live birth history, were not analyzed as independent variables because these data were not uniformly available in the present retrospective dataset. Future prospective studies with larger and more evenly distributed cohorts are needed to validate these findings and improve their generalizability.

## Conclusion

In conclusion, the optimal timing of FET after fresh embryo transfer cancellation may differ according to maternal age. Among women younger than 35 years, undergoing FET within 3 months may be associated with more favorable reproductive outcomes. Among women aged 35–40 years, delaying FET for ≥6 months may be associated with an increased risk of clinical pregnancy loss. These findings support an age-stratified approach to FET timing and may help inform individualized clinical decision-making.

## Data Availability

The datasets generated and/or analyzed during the current study are not publicly available due to privacy and ethical restrictions but are available from the corresponding author on reasonable request.
